# Validation of the patient reported experiences and outcomes of safety in primary care compact Form Brazil

**DOI:** 10.1371/journal.pone.0305414

**Published:** 2024-07-01

**Authors:** Ana Elisa Bauer de Camargo Silva, Tanielly Paula Sousa, Rafael Alves Guimaraes, Valéria Pagotto, Juliana Carvalho de Lima, Maiana Regina Gomes de Sousa, Cristina Alves Bernardes

**Affiliations:** 1 Nursing School, Federal University of Goiás, Goiânia, Brazil; 2 Institute of Tropical Pathology and Public Health, Federal University of Goiás, Goiânia, Brazil; North-West University, SOUTH AFRICA

## Abstract

**Objective:**

To analyze the psychometric properties of the cross-culturally adapted version of the Patient Reported Experiences and Outcomes of Safety in Primary Care (PREOS–PC) Compact Form Brazil.

**Methods:**

A methodological study was conducted with 281 adult Primary Health Care users. Data collection took place online. Confirmatory Factor Analysis (CFA) was used to evaluate the psychometric properties of the PREOS–PC after the process of cross-cultural adaptation to the Brazilian context. Internal consistency was evaluated through Cronbach’s alpha coefficient (α) and McDonald’s omega coefficient (ω).

**Results:**

The sample consisted of 73.3% women. The mean age was 36.1 years (*SD* = 12.2). Of the 23 items of the PREOS–PC that were eligible for CFA, a model with four correlated domains and 16 items presented satisfactory fit indexes. The domains were Practice Activation (PrA) (four items), Patient Activation (PaA) (two items), Experiences of patient safety events (EPaS) (five items) and Outcomes of patient safety (OPaS) (six items). One domain (GPeS) presented one question with a 0 to 10 response scale and two open questions, which cannot be inserted in the CPA due to the nature of the items, but can be included in the application of the scale, being evaluated individually. In this factorial model, five items (EPaS2, EPaS3, EPaS4, EPaS5, EPaS6 and EPaS8) presented factor loadings ≤ 0.30. The α and ω values demonstrated good internal consistency for all domains of the PREOS–PC.

**Conclusions:**

The Brazilian version of the PREOS–PC Compact Form Brazil composed of four domains (PrA, PA, EPaS and OPaS) and 16 items presented evidence of validation of its psychometric properties and can be used to evaluate the experiences and results of patient safety in Primary Health Care in the Brazilian context.

## Background

Patient safety (PS) is conceptualized by the World Health Organization as “the reduction of risk of unnecessary harm associated with healthcare to an acceptable minimum” [1]. Patient safety consists of a set of organized actions that influence healthcare cultures, processes, behaviors, technologies and environments, with the aim of reducing adverse events, errors and their impacts on patients, families, professionals and health services [2].

Most of the research, discussions and efforts that have sought to understand the nature and magnitude of adverse events, evaluating the experiences and results of PS, have been focused on hospital contexts, care environments in which activities are more standardized, complex and carry a higher risk of adverse events [3]. Furthermore, previous research has focused on the experiences and outcomes in the perception of healthcare providers rather than patients [4–6].

Studies that have evaluated these experiences in Primary Health Care (PHC) are limited, especially in relation to the perception of patients [3, 7]. At this level of care, errors related to the use of medications, misdiagnoses and treatments, problems in communication and in the relationship of the healthcare provider with the patients are frequent, which can contribute to increase the magnitude of adverse events [8]. In low- and middle-income countries, the prevalence of PHC safety incidents can vary from 25 to 40% of the care provided, with 80% of these incidents being preventable [9]. Estimates indicate that about 20% and 25% of the general population suffers damage in this scenario in developed and developing countries, respectively [9]. Therefore, unsafe primary care can cause higher morbidity and mortality and increased costs for health services and countries [10].

The experience reported by patients is crucial to improve PHC services, with the aim of improving patient care and promoting the PS culture. This form of evaluation facilitates user involvement, healthcare planning for the improvement and delivery of safe care, increased communication, and trust in healthcare providers [11, 12]. Additionally, patients and their families can assist in the identification of failures in PS or in the quality of care [13].

The use of validated and reliable scales to measure and monitor PS in PHC is indispensable. Some methods are immediately available, easy to administer, do not require external involvement and have low cost [14]. Among them, the Patient Reported Experiences and Outcomes of Safety in Primary Care (PREOS–PC) is a multidimensional instrument developed by specialists and patients that has shown potential for implementation in PHC and use in research. The original instrument, in its final version, has 58 items, with several grouped into five PS domains: a) Practice Activation (PrA), b) Patient Activation (PaA), c) Experiences of patient safety events (EPaS), d) Outcomes of patient safety (OPaS) and e) General perceptions of safety (GPeS) [7].

The PREOSP-PC analyzes the experiences of patients and the results of PS problems in PHC, allowing the differentiation between the different levels of PS between practices and over time in this level of care [7]. It presents three versions, one screening form, one compact form and one comprehensive form, which can be used independently to evaluate five PS domains in PHC in a unique way [15]. The validation study of the comprehensive version was carried out in England with 6,736 patients. Psychometric methods were applied to evaluate its acceptability, reliability, and structural and construct validity, presenting satisfactory parameters [7]. Despite its relevance, this instrument has not yet been cross-culturally adapted and validated for the Brazilian population, which limits its use in the country.

The compact form of the PREOS–PC provides a balance between high psychometric standards and a reduced administrative burden, facilitating its implementation and use in the real practice. Therefore, by validating the scale in PHC care environments in Brazil, it will be possible to identify which experiences are most reported by patients and which PS-related problems need greater attention and, consequently, interventions. The analysis of patients’ experiences may allow an analysis of the current state of PS in PHC, providing relevant data for managers, aiming to develop improvements that meet the needs identified and improve the quality of the services. These actions would have a direct impact on patients’ lives due to the improvement of PS in PHC.

The aim of this study was to analyze the psychometric properties of the cross-culturally adapted version of the PREOS-PC Compact Form Brazil.

## Methods

### Outline

Methodological study to verify the psychometric properties of the PREOS–PC Compact Form Brazil, translated and cross-culturally adapted from the PREOS–PC [7, 16]. Methodological studies deal with the development, validation and evaluation of research tools and methods, such as PREOS–PC Compact Form Brazil.

### Cross-cultural adaptation

The instrument was cross-culturally adapted according to the recommendations of Oxford University Innovation Limited [17] and the methodological guidelines of Beaton et al. [18, 19]. It is important to mention that we obtained the necessary permissions from the owner of the original questionnaire (https://innovation.ox.ac.uk/wp-content/uploads/2018/07/PREOS–PC-27-item-version-SAMPLE.pdf) to carry out the translation and cross-cultural adaptation of the instrument to Brazil.

The process of cross-cultural adaptation of the PREOS–PC Compact Form Brazil was carried out in several stages. First, the questionnaire was translated into Brazilian Portuguese (the language of the participants). The instrument was translated into the target language by two independent native Brazilian translators, with experience in this type of study, one of them with experience in scientific health terminology and the other in linguistics and culture of the Brazilian Portuguese language. The translation of the PREOS–PC instrument into Brazilian Portuguese was carried out in May 2019. Secondly, the synthesis of translations was performed by a third independent bilingual translator, with knowledge and expertise in research in the health area, generating an initial version. This stage was carried out in June 2019. Thirdly, in August 2019, this last version was back-translated by two independent professional translators (with British English as their native language, but fluent in Portuguese), who were not aware of the original version of the assessment instrument or the objectives of the study, which resulted in two more versions.

Fourth, all versions of the instrument were evaluated and approved by an expert committee composed of members of the research team who are experts in the field in question. The review by a committee of experts aimed to evaluate and consolidate all translation and back-translation versions of the instrument, with the aim of developing a single version of the instrument, in the target language, which would be used in the subsequent steps [18].

Finally, a pre-test (pilot) was performed using the final version of the instrument with 30 participants, aiming to evaluate the ability of individuals to interpret and understand the items of the instrument and to provide measures of internal consistency and content validity. This stage was carried out between August 1st and October 30th, 2020. Participants provided consent online, by reading the informed consent form and subsequently accepting the term. After these steps, the participant had access to the questionnaire. The participants in this stage were individuals from two PHC units with a mean age of 42.0 years. The majority (80.0%) were female.

The results of the pre-test showed good understanding of the items by the participants. The McDonald’s omega coefficient (ω) [20] was used to evaluate the internal consistency of the scale, with acceptable values above 0.6. The ω found were 0.689 for the practice activation domain, 0.749 for the patient activation domain and 0.850 for the PS results, demonstrating good internal consistency of the translated and cross-culturally adapted version.

Content validity was also performed by a committee of eight expert judges who evaluated the semantic (verifying whether the words have the same meaning in the original and translated versions), idiomatic (observing expressions difficult to translate that are not suitable for the place of study, to then elaborate an equivalent expression in the target language version), conceptual (verifying whether the words present a different conceptual meaning between cultures) and cultural (trying to find terms that correspond to those described in the instrument and that express situations or activities of daily life in the culture, so that the situations described in the instrument are in accordance with those experienced in the context of the local culture) equivalences of the final instrument [18, 19]. This stage was carried out between November 1st and December 29th, 2020. Each judge evaluated the items through a Likert-type response scale from 1 to 4 (1 = not relevant or not representative, 2 = need for major revision to be representative, 3 = needs minor revision to be representative, 4 = relevant or representative). The mean Content Validity Index (CVI) of 0.96 was calculated, being considered satisfactory (CVI≥0.80) [21].

The participants of the committee of judges were selected from searches on the Lattes Platform of the National Council for Scientific and Technological Development (CNPq) for health professionals with command of Brazilian Portuguese and English; with a master’s or doctorate degree; with experience or work in the area of quality and patient safety or in primary health care; with scientific production and experience in research involving the application of instruments or recognized knowledge in cross-cultural adaptation of measuring instruments, that is, with methodological knowledge in the preparation and/or adaptation of instruments. To avoid regional biases, the diversity of location and place of birth of the judges was considered, which is notably relevant in Brazil, given the large geographic dimension and its regional disparities. An invitation to participate in the committee was sent via email to each specialist. Thus, the committee was made up of eight experts. All judges with specialization in patient safety have already published works on adapting and validating instruments, and only two were not part of a working group related to patient safety.

Of the eight judges, half were nurses, and 7 had a doctorate as their highest degree, in terms of specialty, 4 were specialists in Patient Safety, 3 in measurement/psychometrics and 1 in primary health care. The average time working in the respective specialty areas was 10.25 years. As for the region of Brazil, 4 were from the Central-West, 2 were from the South, 1 was from the Southeast and 1 from the Northeast.

**Fig 1:**
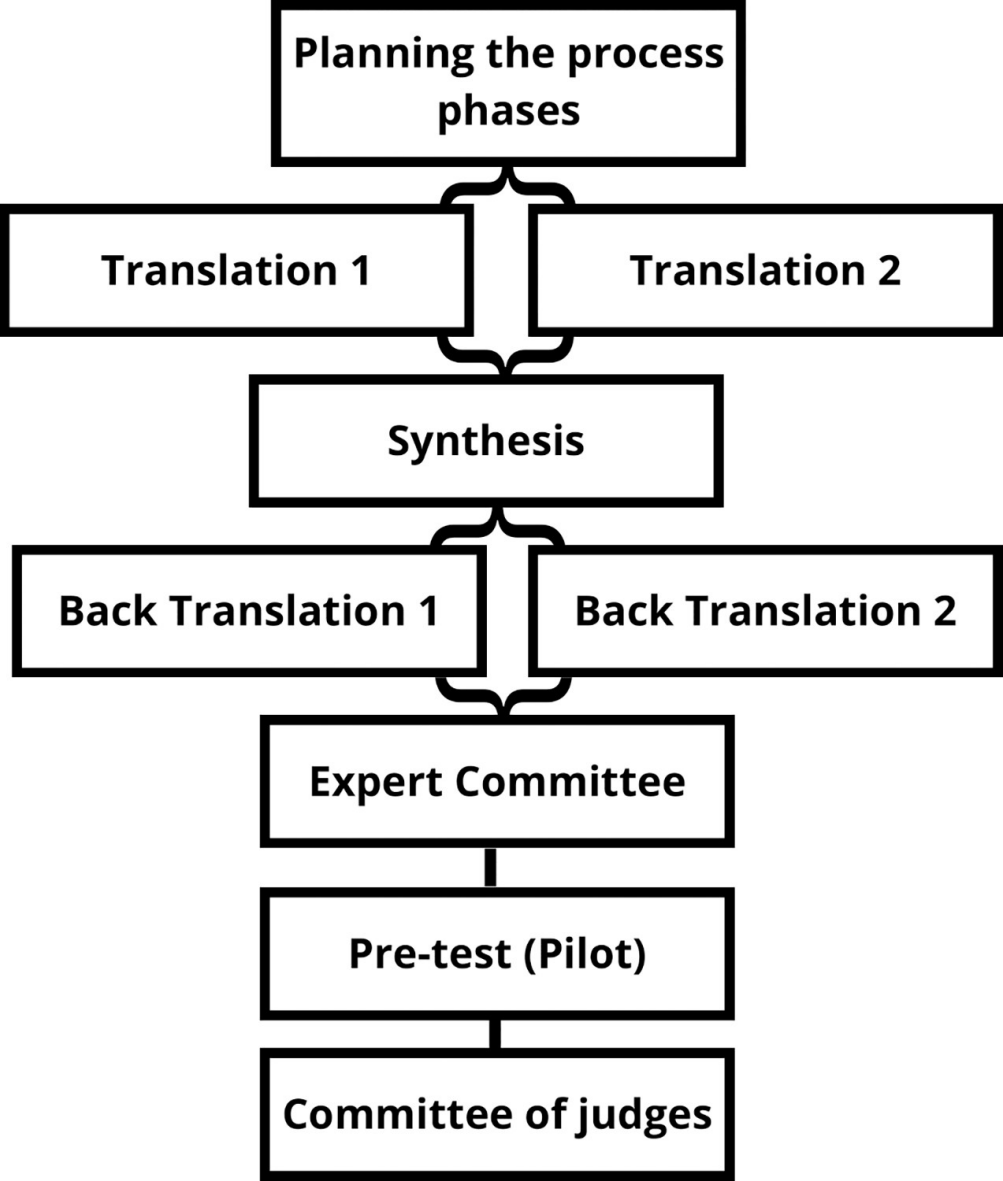
Flowchart of the stages of cross-cultural adaptation.

The flowchart with the stages of cross-cultural adaptation is shown in Fig 1.

The final version of the instrument is available in S1 File.

### Context

The study to evaluate the psychometric properties was conducted in PHC units of Goiânia (estimated population of 1,555,626 inhabitants) [22], located in the state of Goiás, Midwest region of Brazil. All 75 PHC units of the study city were invited to participate in the study, however, only 40 (55.3%) units that agreed. The participating PHC units were Primary Health Units, that perform a set of individual, family and collective health actions that involve health promotion, disease prevention, protection, diagnosis, treatment, rehabilitation, harm reduction, palliative care, and health surveillance, carried out with a multidisciplinary team and directed toward the specific population. All Primary Health Units are considered to be potential spaces for education, training of human resources, research, in-service teaching, innovation, and technological assessment for the health care network [23]. The Primary Health Care coverage in the city at the time of the study was 59.3% [24]. The data was collected between March and July 2021.

### Participants

The population was composed of users of the Brazilian public health system (*Sistema Único de Saúde*–SUS) attended in PHC. Adults aged 18 years or older, of both sexes, native speakers of the Brazilian Portuguese language, registered in one of the 40 units of the PHC network of Goiânia, who had used primary health services at least once in the previous 12 months, regardless of the reason, were included. Individuals without internet connection by mobile phone and/or who did not use the free messaging application WhatsApp were excluded, since the instrument was self-administered via google forms, through a link to access the questionnaire sent through the application.

### Sample size

The minimum sample size was estimated according to the need for five to ten respondents for each item of the instrument [25]. Considering that the PREOS–PC Compact Form is composed of 27 items, a minimum sample size of 270 individuals was required.

### Data collection

Data collection took place through an online access platform. The participants answered two self-administered instruments. The first included the collection of sociodemographic data on health and use of PHC health services in the previous 12 months. This instrument was adapted from questions used and validated in another study [26]. The second included the application of the cross-culturally adapted PREOS–PC Compact Form Brazil.

The selection of participants was performed using the simple random sampling technique, from the list of medical records of the PHC units, where the necessary contact information was obtained, such as the mobile phone number. Due to the pandemic of the disease caused by the SARS-CoV-2 virus (COVID-19), users were contacted by phone and invited to participate in the study. The data collection instruments were also transferred to the Google forms platform and the survey was made available to receive the online responses of the participants through WhatsApp. By accessing the link, the participant was able to view the information about the study and, to proceed, they had to agree to participate in the research. If calls are not answered by the participant, new calls were made to people treated at the same health unit

### Measurements

#### Sociodemographic variables, health conditions and use of health services

The sociodemographic data collected included age (in years), gender (male or female), self-declared race/skin color (white, mixed race, black or other–Asian or indigenous), schooling (up to complete elementary school, incomplete/complete high school or incomplete/complete higher education), family income (minimum wages in reais—R$) (< 1, 1–2 or > 2). Data were also collected on comorbidities (no or yes), use of medications (no or yes), considering the use of any medications as yes [27], and self-assessment health (very bad/bad or good/very good). Data were collected on the use of health services, the number of times they had used the unit’s services in the previous 12 months (1, 2, 3 or ≥4) and the number of consultations (with a doctor and/or nurse) in the unit in the previous 12 months (1, 2–4 or ≥4).

#### PREOS–PC Compact Br

The PREOS–PC Compact Form Brazil was applied to obtain the necessary data for Confirmatory Factor Analysis (CFA).

The instrument presents eight questions and 27 items of different types, including 12 Likert-type items, nine multiple-choice items in which the individual needs to mark one or more items from a checklist of experiences with safety problems, three dichotomous items (yes and no) and two open questions. Question 1 presents four items (PrA1 to PrA4) with Likert-type responses (always, often, sometimes, rarely and never), with the option ‘does not apply’. These items are grouped in the PrA domain. Question 2 presents two items (PaA1 and PaA2) with Likert-type responses (always, often, sometimes, rarely, never), with the option ‘does not apply’. These items are grouped in the PaA domain. Question 3 presents nine multiple-choice items (EPaS 1–9). It should be noted that this question presented an item referring to “None of the above options”, which was excluded from the CFA. Question 4 presents three items (EPaS 10–12), with dichotomous answers (yes and no). The items of question 3 and 4 are grouped in the EPaS domain. Question 5 presents six items (OPaS 1–6) with Likert-type responses (no, yes, some; yes, many; yes, extreme levels), as well as ‘I don’t know’. These items are grouped in the OPaS domain. Finally, question 6, in the GPeS domain, is of the closed type (GPeS1) on a scale from 0 to 10 and questions 7 (GPeS2) and 8 (GPeS3) are open. Questions GPeS1, GPeS2 and GPeS3 were not included in the CFA (Table 1). In the original model, 24 items (excluding items EPaS 9, GPeS1, GPeS2 and GPeS3 due to their response natures) were part of the factorial structure of the scale and were included in the CFA.

**Table 1:** Coding of items and response options of the PREOS–PC Compact Form Brazil.

Codification	Question	Answer options
**Practice activation**		
PrA1	Doctors were available to talk to or assist you when you needed care.	Always, Often, Sometimes, Rarely Never, Does not apply.
PrA2	Doctors encouraged you to talk about concerns or questions you had about your care.	Always, Often, Sometimes, Rarely Never, Does not apply.
PrA3	Doctors have told you which side effects of your treatments you should watch out for (such as feeling sick or diarrhea).	Always, Often, Sometimes, Rarely Never, Does not apply.
PrA4	Doctors took his concerns seriously.	Always, Often, Sometimes, Rarely Never, Does not apply.
**Patient activation**		
PaA1	You told doctors, nurses, or other health professionals at the Basic Health Unit or Family Health Unit when you thought there was something wrong with your care.	Always, Often, Sometimes, Rarely Never, Does not apply.
PaA2	You made a suggestion to doctors, nurses, or other health professionals at the Basic Health Unit or Health Care Unit Family Health when you thought something could be done to improve the service provided.	Always, Often, Sometimes, Rarely Never, Does not apply.
**Experiences of patient safety**		
EPaS1	Diagnosis of your health problem.	Checklist (yes). Otherwise, no.
EPaS2	Medication prescribed or administered to you at your Basic Health Unit or Family Health Unit.	Checklist (yes). Otherwise, no.
EPaS3	Other treatments prescribed or administered to you at your Basic Health Unit or Community Health Unit Family.	Checklist (yes). Otherwise, no.
EPaS4	Vaccines prescribed or administered to you at your Basic Health Unit or Family Health Unit.	Checklist (yes). Otherwise, no.
EPaS5	Blood tests and/or other laboratory tests requested or carried out at your Basic Health Unit or Family Health Unit.	Checklist (yes). Otherwise, no.
EPaS6	Requesting or carrying out other diagnostic and follow-up exams at your Basic Health Unit or Family Health Unit, such as electrocardiogram (ECG), tomography, and X-ray, among others (except blood tests and/or other laboratory tests).	Checklist (yes). Otherwise, no.
EPaS7	Your appointments.	Checklist (yes). Otherwise, no.
EPaS8	Your medical record/file/notes about your care.	Checklist (yes). Otherwise, no.
EPaS9*	None of the above.	Checklist (yes). Otherwise, no.
EPaS10	Communication problem between you and healthcare professionals (for example, not receiving the information you need to take care of your health).	Yes; No
EPaS11	Communication problem between professionals on the healthcare team (for example, not sharing important information about your health or healthcare between professionals in the unit).	Yes; No
EPaS12	Communication problem between health professionals on the unit and health professionals outside the unit (for example, not sharing important information about your health or health care with professionals who work in the specialty outpatient clinic or hospital).	Yes; No
**Outcomes of patient safety (HARM)**		
OPaS1	Harm to your physical health.	Not at all; Yes, some; Yes, a lot; Yes, extreme; I don’t know (yet)
OPaS2	Harm to your mental health.	Not at all; Yes, some; Yes, a lot; Yes, extreme; I don’t know (yet)
OPaS3	Harm that limited your normal social activities (such as see friends or go shopping).	Not at all; Yes, some; Yes, a lot; Yes, extreme; I don’t know (yet)
OPaS4	Harm that increased your health care needs health (such as needing medication or tests).	Not at all; Yes, some; Yes, a lot; Yes, extreme; I don’t know (yet)
OPaS5	Harm that increased your health care needs (such as needing help preparing meals or doing cleaning tasks).	Not at all; Yes, some; Yes, a lot; Yes, extreme; I don’t know (yet)
OPaS6	Harm that led to increased financial expenses with your health.	Not at all; Yes, some; Yes, a lot; Yes, extreme; I don’t know (yet)
**General perceptions of patient safety**		
GPeS1*	On the following scale, assign a score from 0 to 10 for how safe the care you received at the Basic Health Unit or Family Health Unit was in the last 12 months. 0 being completely unsafe and 10 being completely safe.	Scale
GPeS2*	What does the Basic Health Unit or Family Health Unit do effectively to ensure that your health care is safe, that is, without harm to your health or well-being?	Open question
GPeS3*	What suggestions for changes, if any, would you make to your Basic Health Unit or Family Health Unit to ensure that health care is provided safely?	Open question

Note: free translation into English from the instrument validated in the Portuguese version.

*This item is not included in the CFA.

EPaS = Experiences of patient safety; GPeS = General perceptions of patient safety; PaA = Patient activation; PrA = Practice activation; OPaS = Outcomes of patient safety (HARM).

### Statistical analysis

The analyses were performed using the packages “lavaan”, “psych”, “semTools” and “semPlot” in the R language version 4.0.3 [28].

The Anderson-Darling test was initially performed to verify the normality of the quantitative variables. Next, a descriptive analysis of the variables was performed. Quantitative variables were presented as mean and standard deviation. For the PREOS–PC Compact Form Brazil, the median, 25^th^ percentile (P25), 75^th^ percentile (P75), minimum and maximum values were also presented. Qualitative variables were presented as absolute and relative frequency in percentage.

Construct validity was determined using CFA. This statistical technique aims to evaluate the relationship between latent variables (also called domains) and their relations with a pre-established theoretical covariance structure [29]. The Mardia normality test was performed to evaluate the multivariate normality of the PREOS–PC Compact Form Brazil [30]. Due to the absence of multivariate normality (*p*-value < 0.001), a CFA with likelihood estimation with robust variance was performed [31]. Two different factor structures of the PREOS–PC Compact Form Brazil were examined. The initial model of the scale was adjusted through first-order CFA, with four independent domains (PrA, PaA, EPaS, OPaS) and correlated with all 23 eligible items. Next, a final model was established by means of first-order CFA, with four independent and correlated domains, totaling 17 items. In the final model, only items with a factor loading > 0.30 in the initial model remained.

The following indices were used to verify the quality of fit of the models: (i) chi-square fit statistics (χ^2^)–non-significant *p*-values (≥0.05) indicate good quality of fit [32]; (ii) Comparative Fit Index (CFI) and Tucker-Lewis Index (TLI) with acceptable values equal to or greater than 0.95 [33] and (iii) Root Mean Square Error Of Approximation (RMSEA)–values < 0.06 indicate good fit, 0.06–0.07 acceptable fit, 0.08–0.10 limited fit, and > 0.10 inadequate fit [33].

After the estimates, the initial and final models were compared by the Satorra-Bentler Scaled Chi-Square test, which measures the difference between χ^2^ and the degrees of freedom (df) between the original and alternative models. The model chosen, if there was a statistical difference, presented the lowest chi-square rate, in addition to fit indices within the cutoff points [34].

The internal consistency of the instrument was evaluated through Cronbach’s alpha (α) and McDonald’s omega (ω), followed by the respective 95% confidence intervals (95%CI). These coefficients can range from zero to one, and the closer to one, the greater the internal consistency of the instrument. Values of α > 0.70 indicate good internal consistency [35], while values of ω > 0.6 indicate good internal consistency [20].

### Ethical aspects

The study was approved by the Research Ethics Committee of the Clinical Hospital of the Federal University of Goiás, under number 4.298.252/2020. Written informed consent was obtained from all participants.

## Results

A total of 1,650 telephone calls were made and, of these, 979 (59.3%) were answered. Of the total of 979 calls answered, 182 users (18.6%) refused to participate in the study. Thus, of the 797 (81.4%) individuals who agreed to participate in the study, 281 (35.2%) returned the completed questionnaire. Thus, the overall response rate was 28.7% (281/979 calls).

### Characteristics of the participants

The mean age was 36.2 years (SD = 12.2) and the majority (73.3%) were female. The other characteristics of the participants are presented in Table 2.

**Table 2:** Characteristics of participants in the validation stage of the PREOS–PC Compact Form Brazil (n = 281).

Variables		
Sociodemographic		
Age, mean (standard deviation)	36.2 (12.2)	
Sex, n (%)		
Female	206	73.3
Male	75	26.7
Race/skin color (self-declared), n (%)		
White	105	37.4
Mixed-raced	135	48.0
Black	28	10.0
Others (yellow or indigenous)	13	4.6
Education, n (%)		
Up to complete elementary school	62	22.1
Incomplete/complete high school	172	61.2
Incomplete/complete higher education	47	16.7
Income (minimum wages)*, n (%)		
<1	101	35.9
1–2	127	45.2
>2	53	18.9
Health conditions		
Comorbidities, n (%)	122	43.4
Continuous medication use, n (%)	125	44.5
Self-assessment of health, n (%)		
Very bad/bad	47	14.7
Good/very good	234	85.3
Use of health services		
Number of times you used unit services **		
1	64	22.8
2	79	28.1
3	44	15.7
≥4	69	24.6
Missing data	25	8.9
Number of appointments (with doctor and/or nurse)**		
1	56	19.9
2–4	89	31.7
≥4	96	34.2
Missing data	40	14.2

*Minimum wage: R$1.100,00 [1]

**In the last 12 months.

### Descriptive analysis of the PREOS–PC Compact Form Brazil

The Tables 3 and 4 present a descriptive synthesis of the quantitative and qualitative items of the PREOS–PC Compact Form Brazil, respectively.

**Table 3:** Descriptive statistics of the ordinal items (ParA, ParA, SOPaS, GPeS1) of the compact PREOS–PC Compact Form Brazil.

Items	n*	Average (SD)	Median	P25	P75	Min-Max
PrA1	272	3.974 (1.204)	4	3	5	1–5 1–5 1–5 1–5
PrA2	272	3.643 (1.410)	4	3	5	
PrA3	263	3.646 (1.488)	4	3	5	
PrA4	273	4.037 (1.297)	5	3	5	
PaA1	234	3.282 (1.580)	3	2	6	1–5 1–5
PaA2	240	2.783 (1.559)	3	1	4	
OPaS1	269	1.2416 (0.6148)	1	1	1	1–4 1–4 1–4 1–4 1–4 1–4
OPaS2	274	1.2847 (0.6513)	1	1	1	
OPaS3	275	1.1927 (0.5497)	1	1	1	
OPaS4	278	1.3058 (0.693)	1	1	1	
OPaS5	276	1.1884 (0.5663)	1	1	1	
OPaS6	274	1.3321 (0.7674)	1	1	1	
GPeS1	281	7.380 (2.779)	8	6	10	0–10

*Number of valid answers; SD = Standard Deviation; P25 = 25^th^ percentile; P75 = 75^th^ percentile.

**Table 4:** Descriptive statistics of the checklist items, dichotomous items and tight questions of the PREOS–PC Compact Form Brazil.

Items	n*	n**	%
**Experiences of patient safety**			
EPaS1	281	27	9.61
EPaS2	281	9	3.20
EPaS3	281	12	4.27
EPaS4	281	4	1.42
EPaS5	281	12	4.27
EPaS6	281	27	9.61
EPaS7	281	31	11.03
EPaS8	281	15	5.34
EPaS9	281	201	71.53
EPaS10	281	63	22.42
EPaS11	281	55	19.57
EPaS12	281	43	15.30
**General perceptions of patient safety**			
GPeS2			
Access and appointments	281	32	11.39
Patient–centered care	281	65	23.13
Active monitoring	281	17	6.05
Training and technical quality of clinical care	281	9	3.20
Teamwork	281	3	1.07
Environment and equipment	281	45	16.01
Health records	281	2	0.71
Continuity of care	281	8	2.85
Seek patients’ feedback	281	1	0.36
Not reported/Don’t know	281	99	35.23
GPeS3			
Access and appointments	281	88	31.32
Patient–centered care	281	34	12.10
Active monitoring	281	11	3.91
Training and technical quality of clinical care	281	11	3.91
Teamwork	281	7	2.49
Environment and equipment	281	50	17.79
Health records	281	4	1.42
Continuity of care	281	9	3.20
Seek patients’ feedback	281	2	0.71
Not reported/Don’t know	281	65	23.13

*Number of valid responses

**Number of positive responses; SD = Standard Deviation; P25 = 25^th^ percentile; P75 = 75^th^ percentile.

### Confirmatory Factor Analysis

The CFA was conducted to verify the factorial structure of the PREOS–PC Compact Form Brazil. The initial model was adjusted according to a first-order model, with four correlated latent factors (PrA, PaA, EPaS and OPaS) and all 23 eligible items of the PREOS–PC Compact Form Brazil. The “PrA” factor was composed of four items (PrA1 to PrA4), the “PaA” factor of two items (PaA1 and PaA2), the “EPaS” factor encompassed 11 items (EPaS 1 to EPaS12) and the “OPaS” factor included six items (OPaS 1 to OPaS6). This model presented the following quality of fit indices (χ^2^: 327,194; df: 224; *p*-value < 0.001): CFI: 0.908; TLI: 0.897; RMSEA: 0.068 [90%CI: 0.052; 0.085]). The CFI, TLI and RMSEA presented values lower than the cutoff points adopted. In this factorial model, five items (EPaS2, EPaS3, EPaS4, EPaS5, EPaS6 and EPaS8) presented factor loadings ≤ 0.30, which resulted in their exclusion from the final CFA model (Table 5).

**Table 5:** Initial model of the confirmatory factor analysis of the compact PREOS–PC Compact Form Brazil 2021 (n = 281).

Latent variables/items	Estimate	Standard Error	z-value	*p*-value	Factor loadings
**Practice activation**					
PrA1	1.000				0.803
PrA2	1.257	0.086	14.558	<0001	0.873
PrA3	1.272	0.096	13.303	<0001	0.874
PrA4	1.210	0.085	14.319	<0001	0.911
**Patient activation**					
PaA1	1,000				0.879
PaA2	0.906	0.118	7.648	<0001	0.812
**Experiences of patient safety events**					
EPaS1	1.000				0.427
EPaS2	0.054	0.082	0.655	0.512	0.039
EPaS3	0.344	0.191	1.804	0.071	0.211
EPaS4	0.107	0.108	0.991	0.322	0.117
EPaS5	0.207	0.136	1.528	0.127	0.141
EPaS6	0.401	0.237	1.692	0.091	0.175
EPaS7	0.764	0.308	2.481	0.013	0.309
EPaS8	0.435	0.205	2.119	0.034	0.245
EPaS10	2.297	0.556	4.128	<0001	0.728
EPaS11	2.285	0.654	3.493	<0001	0.742
EPaS12	1.781	0.484	3.683	<0001	0.657
**Outcomes of patient safety (HARM)**					
OPaS1	1.000				0.826
OPaS2	0.988	0.087	11.415	<0001	0.816
OPaS3	0.894	0.086	10.376	<0001	0.842
OPaS4	1.152	0.118	9.806	<0001	0.907
OPaS5	1.012	0.123	8.262	<0001	0.892
OPaS6	1.258	0.136	9.279	<0001	0.916
**Covariance**					
Practice activation					
Patient activation	0.443	0.113	3.928	<0001	0.327
Experiences of patient safety events	-0.074	0.025	-2.995	0.003	-0.574
Outcomes of patient safety (HARM)	-0.155	0.051	-3.019	0.003	-0.285
Patient activation					
Experiences of patient safety events (HARM)	0.007	0.016	0.453	0.650	0.040
Outcomes of patient safety (HARM)	0.120	0.053	2.281	0.023	0.157
Experiences of patient safety events					
Outcomes of patient safety (HARM)	0.041	0.014	2.885	0.004	0.554
**Variances**					
PaA1	0.525	0.084	6.223	<0.001	0.355
PaA2	0.473	0.076	6.196	<0.001	0.238
EPaS1	0.479	0.076	6.338	<0.001	0.236
EPaS2	0.289	0.063	4.584	<0.001	0.171
EPaS3	0.564	0.253	2.228	0.026	0.228
EPaS4	0.813	0.244	3.328	0.001	0.341
EPaS5	0.079	0.013	6.002	<0.001	0.818
EPaS6	0.033	0.012	2.797	0.005	0.998
EPaS7	0.045	0.013	3.544	<0.001	0.955
EPaS8	0.014	0.008	1.794	0.073	0.986
EPaS10	0.037	0.012	3.061	0.002	0.980
EPaS11	0.090	0.016	5.555	<0.001	0.969
EPaS12	0.098	0.015	6.341	<0.001	0.905
OPaS1	0.052	0.013	4.009	<0.001	0.940
OPaS2	0.082	0.016	5.168	<0.001	0.469
OPaS3	0.075	0.017	4.434	<0.001	0.449
OPaS4	0.073	0.014	5.305	<0.001	0.568
OPaS5	0.144	0.039	3.704	<0.001	0.318
OPaS6	0.151	0.038	4.023	<0.001	0.334
Practice activation	0.956	0.146	6.558	<0001	1.000
Patient activation	1.915	0.281	6.808	<0001	1.000
Experiences of patient safety events	0.018	0.009	2.069	0.038	1.000
Outcomes of patient safety (HARM)	0.309	0.085	3.624	<0001	1.000

**Fig 2:**
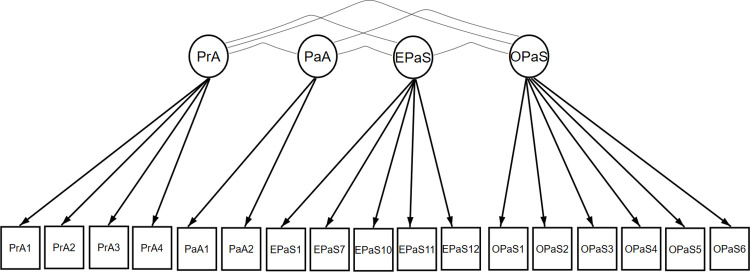
Final model of confirmatory factor analysis of the construct under study of the compact PREOS–PC Compact Form Brazil.

The final model, after excluding the items, was like a first-order model, with four latent factors related to each other (PrA, PaA, EPaS and OPaS), totaling 17 items. The “PrA” factor was composed of four items (PrA1 to PrA4), the “PaA” factor two items (PaA1 and PaA2), the “EPaS” factor five items (EPaS1, EPaS7 and EPaS10 to EPaS12) and the “OPaS” factor six items (OPaS 1 to OPaS6). All items in final model had standardized factor loadings ≥0.3 and were significant, except for item 7 (EPaS7), which had a factorial loading of 0.236, although it was significant. This model had adequate fit indices (χ^2^: 170,850; df: 114; *p*-value < 0.001); CFI: 0.955; TLI: 0.951; RMSEA: 0.050 [90%CI: 0.038; 0.061]), indicating that the factorial structure of the PREOS–PC Compact Form Brazil corresponds to a model with four first-order correlated factors with 17 items (73.9% of the original 23 items). Thus, this model proved to be appropriated, even though item 7 (EPaS7) has a reduced factor loading (Fig 2 & Table 6).

**Table 6:** Final model of the confirmatory factor analysis of the compact PREOS–PC Compact Form Brazil 2021 (n = 281).

Latent variables/items	Estimate	Standard Error	z-value	*p*-value	Factor loadings
**Practice activation**					
PrA1	1.000				0.803
PrA2	1.257	0.086	14.641	<0.001	0.873
PrA3	1.273	0.095	13.369	<0.001	0.874
PrA4	1.212	0.084	14.359	<0.001	0.912
**Patient activation**					
PaA1	1.000				0.883
PaA2	0.896	0.121	7.405	<0.001	0.807
**Experiences of patient safety events**
EPaS1	1.000				0.365
EPaS7	0.685	0.336	2.036	0.042	0.236
EPaS10	2.808	0.785	3.577	<0.001	0.760
EPaS11	2.833	0.875	3.236	0.001	0.786
EPaS12	2.203	0.667	3.303	0.001	0.694
**Outcomes of patient safety (HARM)**
OPaS1	1.000				0.826
OPaS2	0.987	0.087	11.392	<0.001	0.815
OPaS3	0.896	0.087	10.338	<0.001	0.843
OPaS4	1.152	0.118	9.800	<0.001	0.906
OPaS5	1.013	0.123	8.259	<0.001	0.892
OPaS6	1.258	0.136	9.250	<0.001	0.916
**Covariance**					
Practice activation					
Patient activation	0.455	0.097	4.696	<0.001	0.334
Experiences of patient safety events	-0.061	0.023	-2.682	0.007	-0.548
Outcomes of patient safety (HARM)	-0.155	0.051	-3.019	0.003	-0.286
Patient activation					
Experiences of patient safety events (HARM)	0.116	0.045	2.559	0.011	0.150
Outcomes of patient safety					
Experiences of patient safety events	0.030	0.012	2.484	0.013	0.484
Variances					
PrA1	0.527	0.084	6.239	<0.001	0.355
PrA2	0.475	0.076	6.219	<0.001	0.239
PrA3	0.479	0.076	6.333	<0.001	0.236
PrA4	0.286	0.063	4.532	<0.001	0.169
PaA1	0.544	0.262	2.074	0.038	0.220
PaA2	0.829	0.249	3.327	0.001	0.348
EPaS1	0.084	0.014	5.899	<0.001	0.867
EPaS7	0.102	0.016	6.314	<0.001	0.944
EPaS10	0.074	0.016	4.659	<0.001	0.422
EPaS11	0.064	0.016	3.947	<0.001	0.382
EPaS12	0.067	0.014	4.842	<0.001	0.518
OPaS1	0.144	0.039	3.697	<0.001	0.318
OPaS2	0.152	0.038	4.022	<0.001	0.335
OPaS3	0.100	0.032	3.154	0.002	0.289
OPaS4	0.089	0.029	3.090	0.002	0.179
OPaS5	0.081	0.031	2.586	0.010	0.204
OPaS6	0.094	0.025	3.817	<0.001	0.162
Practice activation	0.959	0.145	6.609	<0.001	1.000
Patient activation	1.935	0.289	6.685	<0.001	1.000
Experiences of patient safety	0.013	0.007	1.787	0.074	1.000
Outcomes of patient safety (HARM)	0.308	0.085	3.627	<0.001	1.000

The Satorra-Bentler Scaled Chi-Square test for differences between the initial and final models suggested a better fit of the final model to the factor structure. The *p*-value of the test was 0.0059, which demonstrates that the model with 17 items was significantly higher than the complete model with 23 items.

### Internal consistency

The McDonald ω and Cronbach’s α coefficients ranged from 0.74 (95%CI:0.69–0.80) for the EPaS domain to 0.94 (95%CI:0.92–0.97) for the OPaS domain. The McDonald ω values for the PaA and PrA domains were 0.82 (95%CI: 0.76–0.88) and 0.90 (95%CI = 0.88–0.93), respectively. In each domain, Cronbach’s α values were: EPaS– 0.70 (95%CI: 0.65–0.76), PaA– 0.82 (95%CI: 0.77–0.86), PrA– 0.90 (95%CI: 0.88–0.92) and OPaS– 0.94 (95%CI: 0.93–0.95). The results of the measurements revealed good internal consistency of the PREOS–PC Compact Form Brazil.

## Discussion

The present study provides evidence on the psychometric validity of the PREOS–PC Compact Form Brazil with a sample of PHC patients. This instrument has been used in other countries, but, to date, its validation had not been performed and its applicability remains limited in the Brazilian context. The results demonstrate the validity of the PREOS–PC Compact Form Brazil, with a factorial structure composed of 17 items and four correlated domains (PrA, PaA, EPaS and OPaS). The instrument presented good internal consistency. Therefore, it represents evidence of construct validity to measure PS perceptions, experiences and results from the perspective of PHC users.

The initial model (with 23 items and four correlated factors) presented low factorial validity indexes. However, after the exclusion of six items, the model proved to be adequate, with good fit indexes. The excluded items were EPaS2, EPaS3, EPaS4, EPaS5, EPaS6 and EPaS7, all dichotomous and belonging to the EPaS domain. They relate to a single question that covers the problems that patients may have faced during PHC care. The inclusion of dichotomous items in the CFA model may result in biased parameter estimates with inaccurate interpretation limitations [36]. Therefore, the absence of contribution to the domains of these items may have been caused by the very nature of the items and, not necessarily, because they did not contribute to the formation of the construct. Another point is that some excluded items relate to health practices that are uncommon in the context of PHC in Brazil, particularly the administration of medications in the health unit, treatments, blood tests, laboratory tests and diagnostic tests, which are predominantly performed in the secondary (emergency and urgency units) and tertiary (for example: hospitals) networks [37]. This can decrease the probability of the patient’s knowledge about aspects related to the problems faced during their PHC care. All the excluded items, in fact, presented low prevalence in the sample, which may have contributed to their non-fit in the domains. In addition, the collection was carried out in the context of the COVID-19 pandemic in a scenario of reduced supply of health services [38], which may have contributed to the low prevalence of these items.

The final CFA model showed four correlated domains: “PrA”, “PaA”, “EPaS” and “OPaS”. The first contains items that evaluate what the health unit does to create a safe environment and ensure PS. The second presents items that seek to understand how patients are proactive to ensure that the healthcare is safe. The third has items that assess the prevalence of safety errors related to healthcare. The fourth presents items that assess the presence of health damage experienced by users. A domain that is not adjusted to the CFA due to the nature of the items, is called “General perceptions of patient safety”, which aims to evaluate how safe patients perceive their health unit to be and should be analyzed in isolation [26].

Internal consistency was assessed using Cronbach’s α and McDonald’s ω. The values were adequate, higher than recommended (α > 0.70 and ω > 0.60) [20, 35]. McDonald’s ω is a measure of solid internal consistency, but is little used in scale validation studies [39]. The PREOS–PC is relatively recent. No studies were found that analyzed the internal consistency of the instrument, along with the psychometric properties of the compact version. To date, the reliability of the instrument has only been tested for the full version, using Cronbach’s α, but not McDonald’s ω. [7]. The results of the full version study showed high internal consistency for the domains (Cronbach’s α: PrA = 0.89; PaA = 0.80, EPaS = 0.75 and OPaS = 0.96) [7]. Although with differences in the items, the internal consistencies were similar in the present study (Cronbach’s α: PrA = 0.90; PaA = 0.82, EPaS = 0.74 and OPaS = 0.94). In the original study, the EPaS domain presented the lowest internal consistency value, which is like that found in this study, which may explain the removal of some of the items from the final model. It is important to note that the PREOS–PC Compact Form Brazil is a reduced version and, to date, there are no studies that can accurately compare this version, since the original extended instrument has 58 items [7], which prevents a more accurate comparison with the literature.

The validity of the structural construct of the General Perceptions of Patient Safety (GPeS) domain was not confirmed by factor analysis, since one item was composed of a scale from 0 to 10 and two were open questions. Therefore, the compact version does not allow a factorial evaluation of the instrument, since it does not include this domain. It is important to note that, in the full version, the “General perceptions of patient safety” domain presents a single scale question and two open questions [7].

The present study has some limitations. Data collection was performed in a single Brazilian city, which limits the ability to generalize the results. The experiences and reports of the patients in these PHC units may not be the same as the practice in the rest of the country. The study sample was composed of 73.3% women, which does not guarantee that the results found are not distorted by the lack of sampling balance between the sexes, and may not be generalized to men [40]. Additionally, the data was collected online, due to the COVID-19 pandemic. These individuals may have characteristics that differ from people who do not have access to the internet, which also limits generalization. In addition, the validation study of the full version was performed through the application of the physical instrument. Some calls went unanswered, which may have led to selection bias. All measures were self-reported, susceptible to memory and response biases. The inclusion of dichotomous items in the CFA is another limitation, which reduces the contribution of these items in the latent domain.

However, the study has strengths. First, the relatively large sample size allowed the inclusion of enough patients to conduct CFA. Second, this psychometric validation study is unprecedented in Brazil, being a starting point for future investigations that may be carried out in other Brazilian regions, as well as the implementation of the instrument in the PHC practice of the country. The relevance of this study is the possibility of improving the quality of care in PHC in Brazil, adapting methodologies to PS perceptions, experiences and results, through the evaluation of the patients themselves. This study can also help in the implementation of a process to improve the PHC care environment, as well as consolidate knowledge about the theme in the literature. However, further studies are still needed to evaluate the psychometric properties of the PREOS–PC Compact Form Brazil, especially in relation to some of the less prevalent items. Accordingly, future studies with more diverse samples should be carried out to have a clear understanding of users’ perceptions about PS in PHC, so that other comparisons and generalizations can be made. It is important to emphasize that, especially, conducting studies in other Brazilian regions, in a self-administered and face-to-face manner, could contribute to the improvement of the instrument and to the identification of new tests of validity and reliability for the PREOS–PC Compact Form Brazil in different contexts.

## Conclusions

The Brazilian version of the PREOS–PC Compact Form Brazil presented satisfactory validity and psychometric properties for 16 items and four domains (PrA, PaA, EPaS and OPaS). The items EPaS2, EPaS3, EPaS4, EPaS5, EPaS6 and EPaS7 presented low factor loadings, which indicates that, in the Brazilian scenario, they do not contribute to the latent variables of the domains. However, these items should be incorporated into future psychometric studies for further assessment. Furthermore, the isolated analysis of the GPeS domain items is not suitable for CFA but should be considered when assessing PS in PHC. The instrument showed good internal consistency.

## Supporting information

S1 File Final version of the Patient Reported Experiences and Outcomes of Safety in Primary Care Compact Form Brazil (PREOS–PC Compact Form Brazil), in Portuguese. (PDF)

S2 File Dataset. (XLSX)

## Abbreviations

CFA: Confirmatory Factor Analysis
PHC: Primary Health Care
CFI: Comparative Fit Index
COVID-19: disease caused by the SARS-CoV-2 virus
EPaS: Experiences of patient safety events
df: Degrees of Freedom
GPeS: General perceptions of safety
90%CI: 90% Confidence Interval
95%CI: 95% Confidence Interval
CVI: Content Validity Index
OPaS: Outcomes of patient safety
PaA: Patient Activation
PrA: Practice Activation
PREOS–PC: Patient Reported Experiences and Outcomes of Safety in Primary Care
R$: Brazilian Reais
RMSEA: Root Mean Square Error of Approximation
PS: Patient Safety
SUS: Brazilian National Health System
TLI: Tucker-Lewis Index
α: Cronbach’s alpha
χ^2^: Chi-square goodness-of-fit test statistic
ω: McDonald’s omega
