# Supplementary material for: Validation of the patient reported experiences and outcomes of safety in primary care compact Form Brazil
Source: PLoS One. 2024 Jul 1;19(7):e0305414. doi: 10.1371/journal.pone.0305414 (PMC11216591; doi:10.1371/journal.pone.0305414)
Supplement: S1 File — (PDF) [file pone.0305414.s001.pdf]

Relatos de Pacientes sobre Experiências e Resultados de Segurança do Paciente na Atenção Primária à saúde - PREOS-PC Compacto Versão Brasil

Sua experiência de atendimento na Unidade Básica de Saúde e Unidade de Saúde da Família

Sobre o que é esta pesquisa?

Pacientes recebem atendimento nas Unidades Básicas de Saúde e Unidades de Saúde da Família no Brasil, que, geralmente, é seguro e de qualidade. No entanto, este atendimento pode, às vezes, ser prejudicial à saúde ou ao bem-estar do paciente.

Por exemplo:

- Você pode não ser examinado ou não receber um exame/teste quando necessário;
- Você pode receber um diagnóstico errado; ou
- Podem lhe dar o medicamento errado;

Gostaríamos que você respondesse a essa pesquisa para termos informações sobre como melhorar a qualidade e a segurança dos serviços na Unidade Básica de Saúde ou Unidade de Saúde da Família onde você é atendido(a).

Sua opinião é muito importante para ajudar a melhorar a qualidade dos atendimentos prestados e a segurança dos pacientes.

Por favor, participe desta pesquisa mesmo que você nunca tenha tido problemas ao ser atendido(a) na Unidade Básica de Saúde ou Unidade de Saúde da Família. Esta pesquisa deve levar cerca de 10 minutos para ser preenchida.

Por favor, leia as instruções atentamente antes de começar a responder a pesquisa.

Como preencher a pesquisa:

- Por favor, selecione, a seguir, o item que corresponde à sua resposta. Caso você esteja respondendo no formato online, clique nas respostas desejadas; se estiver respondendo no formato impresso, marque um "X" nas suas respostas.

- Não se preocupe se cometer um erro ou mudar de ideia. Em caso de preenchimento da pesquisa no formato online, basta selecionar uma nova resposta, e a sua primeira opção será desmarcada. Em caso de preenchimento da pesquisa no formato impresso, basta preencher totalmente a caixa que você havia marcado (desta forma: ■) e coloque um X na nova caixa.

- Se você é um cuidador e está respondendo a pesquisa em nome de alguém, por favor, responda a respeito do atendimento de saúde prestado à pessoa que você está cuidando, em vez de considerar o atendimento que você recebeu na Unidade Básica de Saúde ou Unidade de Saúde da Família.

- Seu nome não será usado na pesquisa. Ela é anônima. Portanto, não será feita nenhuma notificação a qualquer órgão público ou instituição em seu nome. Se você precisar de orientação sobre qualquer problema relacionado à segurança do cuidado recebido na Unidade Básica de Saúde ou Unidade de Saúde da Família, por favor, entre em contato com a unidade de saúde ou serviço de ouvidoria de saúde.

Obrigado por sua disponibilidade de tempo e ajuda!

1. Considerando o atendimento que você recebeu na Unidade Básica de Saúde ou Unidade de Saúde da Família nos últimos 12 meses, com que frequência os seguintes itens se aplicaram aos seus médicos?

	Sempre	Frequentemente	Às vezes	Raramente	Nunca	Não se Aplica
Os médicos estavam disponíveis para conversar ou lhe atender quando você precisava ser atendido	☐	☐	☐	☐	☐	☐
Os médicos estimulavam você a conversar sobre preocupações ou dúvidas que tivesse sobre seu atendimento	☐	☐	☐	☐	☐	☐
Os médicos falaram sobre quais efeitos colaterais dos seus tratamentos você deveria observar (tais como mal-estar ou diarreia)	☐	☐	☐	☐	☐	☐
Os médicos levaram suas preocupações a sério	☐	☐	☐	☐	☐	☐

2. Considerando o atendimento de saúde que você recebeu na Unidade Básica de Saúde ou Unidade de Saúde da Família nos últimos 12 meses, com que frequência você fez as seguintes ações?

	Sempre	Frequentemente	Às vezes	Raramente	Nunca	Não se Aplica
Você contou aos médicos, enfermeiros ou outros profissionais de saúde da Unidade Básica de Saúde ou Unidade de Saúde da Família quando você achou que havia algo errado no seu atendimento	☐	☐	☐	☐	☐	☐
Você fez uma sugestão aos médicos, enfermeiros ou outros profissionais de saúde da Unidade Básica de Saúde ou Unidade de Saúde da Família quando você achou que algo poderia ser feito para melhorar o atendimento prestado	☐	☐	☐	☐	☐	☐

A seguir, gostaríamos de perguntar se você teve algum problema no seu atendimento na Unidade Básica de Saúde ou Unidade de Saúde da Família que possa ter prejudicado a sua saúde ou o seu bem-estar como, por exemplo, não ser examinado, não ser tratado quando necessário ou receber um diagnóstico errado ou medicamento errado.

A próxima pergunta contém uma lista de problemas de segurança que podem acontecer no seu atendimento em Unidade Básica de Saúde ou Unidade de Saúde da Família.

3. Considerando o atendimento que você recebeu na sua Unidade Básica de Saúde ou Unidade de Saúde da Família nos últimos 12 meses, houve algum problema que possa ter prejudicado a sua saúde ou bem-estar relacionado às seguintes situações? (Por favor, marque as situações que ocorreram problemas).

Diagnóstico do seu problema de saúde.	☐
Medicamento prescrito ou administrado à você na sua Unidade Básica de Saúde ou Unidade de Saúde da Família.	☐
Outros tratamentos prescritos ou administrados à você na sua Unidade Básica de Saúde ou Unidade de Saúde da Família.	☐
Vacinas prescritas ou administradas à você na sua Unidade Básica de Saúde ou Unidade de Saúde da Família.	☐
Exames de sangue e/ou outros exames laboratoriais solicitados ou realizados na sua Unidade Básica de Saúde ou Unidade de Saúde da Família.	☐
Solicitação ou realização de outros exames de diagnóstico e acompanhamento na sua Unidade Básica de Saúde ou Unidade de Saúde da Família como, por exemplo, eletrocardiograma (ECG), tomografia, raio-X, dentre outros (exceto exames de sangue e/ou outros exames laboratoriais)	☐
Suas consultas	☐
Seu prontuário / ficha / anotações sobre seu atendimento	☐
Nenhuma das opções acima	☐

4. Considerando o atendimento que você recebeu na Unidade Básica de Saúde ou Unidade de Saúde da Família, nos últimos 12 meses, houve algum dos seguintes problemas de comunicação?

	Sim	Não
Problema de comunicação entre você e os profissionais de saúde (por exemplo, não receber as informações de que precisava para cuidar de sua saúde)	☐	☐
Problema de comunicação entre os profissionais da equipe de saúde (por exemplo, não compartilhamento de informações importantes sobre sua saúde ou cuidados de saúde entre os profissionais da unidade)	☐	☐
Problema de comunicação entre profissionais de saúde da unidade com profissionais de saúde que não são da unidade (por exemplo, não compartilhamento de informações importantes sobre sua saúde ou cuidados de saúde com profissionais que atuam no ambulatório de especialidades ou no hospital)	☐	☐

A seguir, gostaríamos de perguntar se você sofreu algum dano como resultado dos cuidados recebidos na Unidade Básica de Saúde ou Unidade de Saúde da Família.

Por dano, nos referimos a situações em que o atendimento em si causa um problema para a saúde ou o bem-estar do paciente.

Às vezes, isto ocorre porque o atendimento não é tão bom quanto deveria. Por exemplo, um paciente com sintomas de câncer que necessita de uma investigação aprofundada, é atendido por um médico que não reconhece a importância dos sintomas e não toma uma ação. Meses depois, o câncer é diagnosticado, mas em um estágio mais avançado do que quando foi atendido pela primeira vez.

O dano também pode ocorrer mesmo com um cuidado de alta qualidade, por exemplo, quando um paciente recebe o medicamento correto, mas desenvolve uma reação inesperada que o faz se sentir mal.

Por favor, responda a próxima pergunta (5) mesmo se você respondeu nas questões 3 e 4 que não teve nenhum problema de segurança que possa ter levado a algum prejuízo à sua saúde ou bem-estar nos últimos 12 meses.

5. Você acha que teve algum dos seguintes tipos de dano como resultado dos cuidados oferecidos na Unidade Básica de Saúde ou Unidade de Saúde da Família nos últimos 12 meses?

	Não	Sim, alguns	Sim, muitos	Sim, em níveis extremos	Não sei dizer
Dano à sua saúde física	☐	☐	☐	☐	☐
Dano à sua saúde mental	☐	☐	☐	☐	☐
Dano que limitou suas atividades sociais normais (como ver amigos ou fazer compras)	☐	☐	☐	☐	☐
Dano que aumentou suas necessidades de cuidados de saúde (como precisar de medicação ou de exames)	☐	☐	☐	☐	☐
Dano que aumentou suas necessidades de cuidados de saúde (como precisar de ajuda para preparar as refeições ou fazer tarefas de limpeza)	☐	☐	☐	☐	☐
Dano que levou ao aumento dos gastos financeiros com a sua saúde	☐	☐	☐	☐	☐

6. Na escala a seguir, atribua uma nota de 0 a 10, para o quão seguro foi o atendimento que você recebeu na Unidade Básica de Saúde ou Unidade de Saúde da Família nos últimos 12 meses. Sendo 0 completamente inseguro e 10 completamente seguro.

0 1 2 3 4 5 6 7 8 9 10

Completamente inseguro **Completamente seguro**

7. O que a Unidade Básica de Saúde ou Unidade de Saúde da Família faz de forma eficaz para garantir que o atendimento à sua saúde seja seguro, ou seja, sem prejuízo à sua saúde ou bem- estar?

____
____
____
____
____
____

8. Quais sugestões de mudanças, se houver, você daria à sua Unidade Básica de Saúde ou Unidade de Saúde da Família para assegurar que o atendimento à saúde seja fornecido com segurança?

____
____
____
____
____
____

Obrigado por sua disponibilidade de tempo e ajuda!
